# The Specific IKKε/TBK1 Inhibitor Amlexanox Suppresses Human Melanoma by the Inhibition of Autophagy, NF-κB and MAP Kinase Pathways

**DOI:** 10.3390/ijms21134721

**Published:** 2020-07-02

**Authors:** Moritz Möller, Julia Wasel, Julia Schmetzer, Ulrike Weiß, Markus Meissner, Susanne Schiffmann, Andreas Weigert, Christine V. Möser, Ellen Niederberger

**Affiliations:** 1 Pharmazentrum frankfurt/ZAFES, Institute of Clinical Pharmacology, Faculty of Medicine, Goethe-University Frankfurt, Theodor Stern Kai 7, 60590 Frankfurt am Main, Germany; m_moeller@mail.de (M.M.); julia.wasel@arcor.de (J.W.); juliasch@yahoo.de (J.S.); weiss@med.uni-frankfurt.de (U.W.); chmoeser@hotmail.com (C.V.M.); 2 Department of Dermatology, Venereology and Allergology, Faculty of Medicine, Goethe-University Frankfurt, Theodor Stern Kai 7, 60590 Frankfurt, Germany; Markus.Meissner@kgu.de; 3 Fraunhofer Institute for Molecular Biology and Applied Ecology (IME), Branch for Translational Medicine and Pharmacology TMP, Theodor Stern-Kai 7, 60590 Frankfurt am Main, Germany; Susanne.Schiffmann@ime.fraunhofer.de; 4 Institute of Biochemistry I, Faculty of Medicine, Goethe-University Frankfurt, Theodor Stern Kai 7, 60590 Frankfurt am Main, Germany; weigert@biochem.uni-frankfurt.de

**Keywords:** melanoma, IKKε, TBK1, amlexanox, tumor growth, NF-кB

## Abstract

Inhibitor-kappaB kinase epsilon (IKKε) and TANK-binding kinase 1 (TBK1) are non-canonical IκB kinases, both described as contributors to tumor growth and metastasis in different cancer types. Several hints indicate that they are also involved in the pathogenesis of melanoma; however, the impact of their inhibition as a potential therapeutic measure in this “difficult-to-treat” cancer type has not been investigated so far. We assessed IKKε and TBK1 expression in human malignant melanoma cells, primary tumors and the metastasis of melanoma patients. Both kinases were expressed in the primary tumor and in metastasis and showed a significant overexpression in tumor cells in comparison to melanocytes. The pharmacological inhibition of IKKε/TBK1 by the approved drug amlexanox reduced cell proliferation, migration and invasion. Amlexanox did not affect the cell cycle progression nor apoptosis induction but significantly suppressed autophagy in melanoma cells. The analysis of potential functional downstream targets revealed that NF-кB and ERK pathways might be involved in kinase-mediated effects. In an in vivo xenograft model in nude mice, amlexanox treatment significantly reduced tumor growth. In conclusion, amlexanox was able to suppress tumor progression potentially by the inhibition of autophagy as well as NF-кB and MAP kinase pathways and might therefore constitute a promising candidate for melanoma therapy.

## 1. Introduction

Malignant melanoma is the most aggressive type of skin cancer, with increasing global incidence. It has a highly metastatic potential, and the 5-year survival rates of patients with advanced melanoma are very poor. The therapy of malignant melanoma is mostly based on the resection of the tumor. Pharmacological treatment is very difficult, and responses are highly variable [1]. Melanoma are often characterized by the hyperactivation of the RAS/RAF/MEK/MAPK signaling cascade as a consequence of different oncogenic mutations. Common variants comprise BRAF (e.g., BRAF^V600^) or NRAS, both involved in mitogen activated protein kinase (MAPK) activation, as well as the tumor suppressor phosphatase and tensin homolog (PTEN), respectively. These mutations contribute to the highly proliferative and pro-survival properties of melanoma cells and have raised opportunities for targeted therapies of melanoma patients; however, several melanoma quickly develop resistance against their respective drugs [2]. This is at least partially due to the low susceptibility of melanoma cells against apoptosis induction. On the other hand, basal autophagy is increased in melanoma cells and a high autophagic flux is reported in melanoma patients. The increase in autophagy has also been associated with a reduced drug efficacy (reviewed in [3]). Therefore, there is a great need for the development of novel therapeutic approaches based on the so-far unknown molecular mechanisms of melanoma initiation and progression.

The inhibitor-kappaB kinase (IKK)-related kinases IKKε and TANK-binding kinase (TBK)1 are involved in the activation of the transcription factor nuclear factor kappa B (NF-кB) by the phosphorylation of components of its activation cascade [4,5,6,7,8]. The dysregulations of NF-кB signaling contribute to a variety of different tumors, including melanoma [9] -e.g., human skin cancers show an increased NF-кB expression and nuclear translocation in comparison to naevi and healthy skin [10,11]. Furthermore, the increased activity of the classical IKKs α and β has been described in malignant melanoma, resulting in a constitutive activation of NF-кB [12]. IKKε and TBK1 are also associated with the initiation and progression of several cancer types (reviewed in [13,14]). Both kinases are able to phosphorylate the tumor suppressor gene cylindromatosis (CYLD) and thereby inhibit its function [15]. This effect has contributed to the description of IKKε as an oncogene which could be responsible for malignant transformation and carcinoma metastasis by directly or indirectly phosphorylating and thereby activating NF-кB p65 [4,16,17]. In breast and prostate cancer cell lines, the silencing of IKKε resulted in the reduced activation of NF-кB and inhibition of cell proliferation. Consequently, a knockdown of IKKε in vitro resulted in the significant inhibition of breast and pancreatic cancer cell proliferation and survival [18,19]. Similar effects were shown for TBK1 in KRAS-driven cancers [20]. TBK1 has already been interconnected with special subsets of melanoma [21,22], while the function of IKKε in the initiation and progression of human melanoma is not known so far. In a previous study, we could show that IKKε is overexpressed in murine malignant melanoma cells in comparison to melanocytes. The inhibition of IKKε in tumor cells was associated with reduced cell proliferation and resulted in the altered regulation of the NF-кB, Akt1 and MAPK pathways. The inoculation of IKKε-depleted melanoma cells in wild-type mice resulted in a significantly reduced tumor development as compared to wild-type melanoma cells [23]. In another study, IKKε-depleted mice showed a higher survival rate and less tumor development than wild-type mice after a systemic injection of melanoma cells, probably due to an increase in T-cell-mediated anti-tumor immunity [24]. These results indicated that TBK1 and IKKε are involved in the pathophysiology of melanoma and might therefore represent suitable targets for novel therapeutic approaches. In this study, we investigated the expression of IKKε and TBK1 in human melanoma cells, as well as in human tissue samples comprising naevi, primary melanoma and melanoma metastasis. Furthermore, we assessed the effect of the selective TBK1/IKKε inhibitor amlexanox on tumor cell growth, migration and invasion in cell culture and in an in vivo melanoma xenograft model in nude mice.

## 2. Results

### 2.1. IKKε and TBK1 Expression in Human Melanocytes and Melanoma Cells

**Figure 1:**
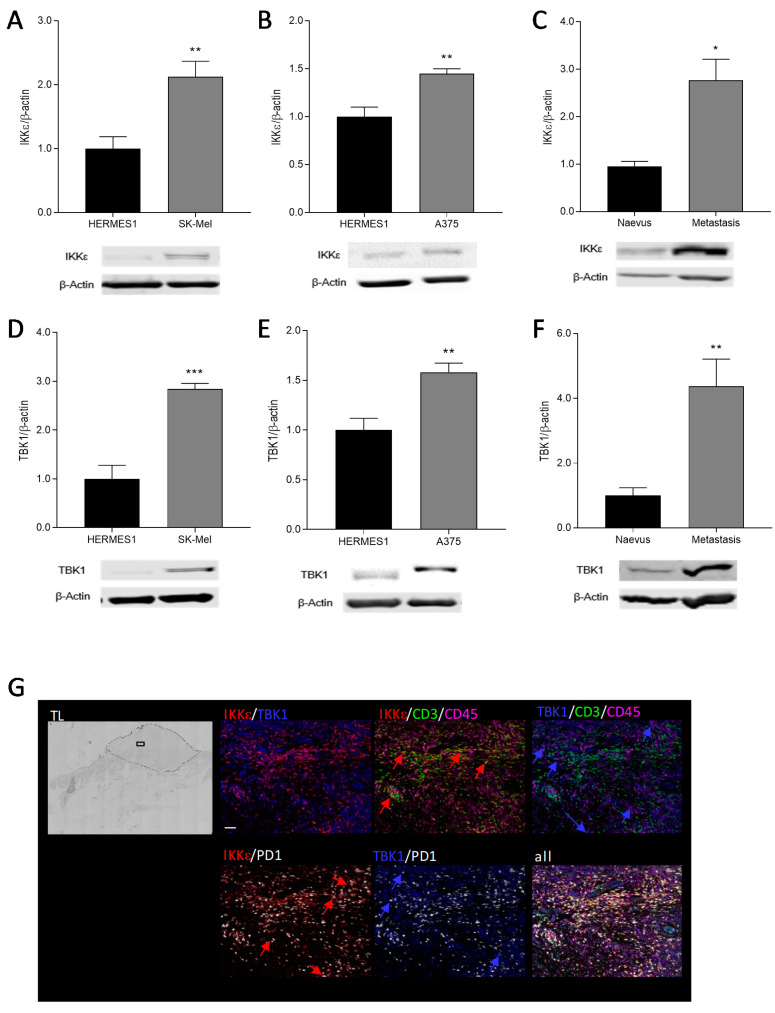
IKKε and TBK1 protein expression in human cells and tissues. (**A**) IKKε protein expression in human extended-replication *melanocytes* (HERMES)1 (black) and SK-Mel-28 melanoma cells (dark grey) (*n* = 5–7), (**B**) in HERMES1 (black) and A375M melanoma cells (dark grey) (*n* = 4) and (**C**) in human tissue from healthy naevi (black) in comparison to melanoma metastasis (dark grey) (*n* = 9–12). (**D**) TBK1 protein expression in human HERMES1 melanocytes (black) and SK-Mel-28 melanoma cells (dark grey) (*n* = 6), (**E**) in HERMES1 (black) and A375M melanoma cells (dark grey) (*n* = 8) and (**F**) in human tissue from healthy naevi (black) in comparison to melanoma metastasis (dark grey) (*n* = 12). The Western blots show one representative blot, the bar diagrams show the densitometric analysis of all blots. * *p* < 0.05, ** *p* < 0.01, *** *p* < 0.001. (**G**) Multiplex immunofluorescence in paraffin-embedded primary melanoma sections. Representative image out of 5 different patient samples (IKKε (red), TBK1 (blue), CD45 (magenta), CD3 (green), PD1 (white)). Scale Bar: 100 µM. TL is a transmitted light overview picture of the slice (magnification 4×); the dotted line shows the tumor, the square indicates the enlarged region. Blue arrows indicate the colocalization of TBK1 with a respective marker protein; red arrows point out the colocalization of IKKε with a respective marker protein.

To gain insight into the potential functional relevance of IKKε and TBK1 in melanoma, we assessed the expression levels of IKKε and TBK1 in two different human melanoma cell lines in comparison with melanocytes. A Western blot analysis revealed a relatively low expression of both IKKε and TBK1 proteins in human HERMES1 melanocytes, which were significantly elevated in A375M and SK-Mel-28 melanoma cells (Figure 1a,b,d,e). These data were supported by expression analyses using tissue samples from melanoma patients and healthy donors. Melanoma metastasis revealed a significantly higher protein expression of IKKε and TBK1 in comparison to naevi (Figure 1c,f), thus further hinting at a relevant role of IKKε and TBK1 in the pathophysiology of skin cancer. In slices of primary human melanoma, we performed a multiplex immunofluorescence analysis and observed the expression of both IKKε and TBK1 in pigmented tumor cells in the melanoma. IKKε showed a strong colocalization with CD3-positive cells, while TBK1 colocalized partially with CD45- but not with CD3-positive immune cells. Several of the TBK1 and IKKε-stained cells showed additional immunoreaction with the immune inhibitory receptor PD1 (Figure 1g).

### 2.2. Proliferation of Melanoma Cells after Inhibition of Ikkε/TBK1 by Amlexanox

**Figure 2:**
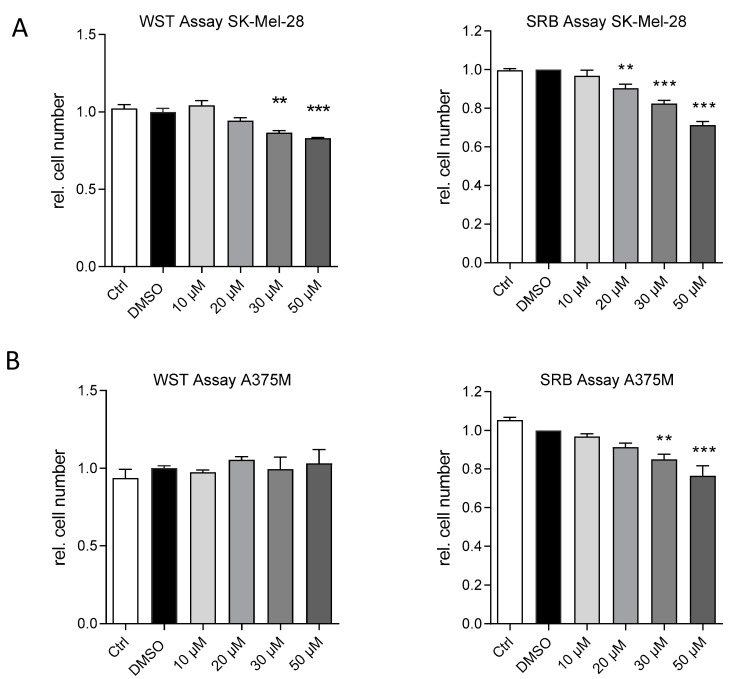
Effects of amlexanox on cell proliferation and cytotoxicity. The cell number and the proliferation rate were assessed using the SRB cytotoxicity (right panel) and the WST cell proliferation assay (left panel), respectively. (**A**) SK-Mel-28 cells were treated for 48 h with 0–50 µM amlexanox or 0.3% DMSO as a negative control. Untreated SK-Mel-28 cells served as an additional control. (**B**) A375M cells were treated for 48 h with 0–50 µM amlexanox or 0.3% DMSO as a negative control. Untreated A375M cells served as an additional control. Experiments were independently repeated at least three times (*n* = 3–9). ** *p* < 0.01, *** *p* < 0.001 in comparison to vehicle-treated control cells.

The proliferation of A375M and SK-Mel-28 human melanoma cells was examined after the incubation of the cells with increasing concentrations of the IKKε/TBK1 small molecule inhibitor amlexanox. The sulforhodamine B (SRB) assay delivers hints for the cytotoxic activity of drugs, while the water soluble tetrazolium (WST) metabolism is directly proportional to the cell proliferation activity. The results of both assays indicate that the vehicle (0.3% dimethylsulfoxide (DMSO)) as well as low concentrations of amlexanox have no impact on cell proliferation. However, in SK-Mel-28 cells, a significant antiproliferative effect of amlexanox was observed at concentrations higher than 20 µM in the WST test and at concentrations above 10 µM in the SRB assay (Figure 2a). The calculated inhibitory concentration (IC)_50_ values were 92 µM in the SRB and 125 µM in the WST assay, respectively. The A375M cells were not affected by amlexanox in the WST test. In the SRB assay, we observed significant reductions in the cell number starting at concentrations above 30 µM (Figure 2b). The calculated IC_50_ value was 117 µM. These results support the assumption that IKKε and TBK1 are involved in the proliferation and survival of melanoma cells. Since the effects were clearer and more stable with SK-Mel-28 melanoma cells, all further experiments were performed preferentially with this cell line.

### 2.3. Potential Mechanisms Contributing to the Decreased Cell Proliferation after the Inhibition of IKKε/TBK1 by Amlexanox

**Figure 3:**
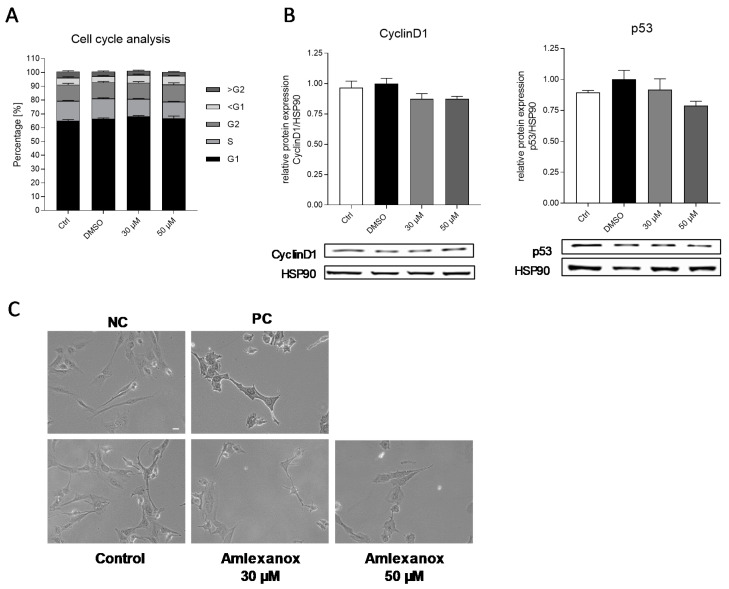
Cell cycle analysis and apoptosis induction in SK-Mel-28 cells. (**A**) Fluorescence activated cell sorting (FACS) analysis of SK-Mel-28 cells. Cells were synchronized in the G1 phase of the cell cycle, then incubated with amlexanox for 48 h and, subsequently, the percentage of cells in different cell cycle phases was analyzed (*n* = 3). (**B**) Western blot analysis of cyclinD1 and p53 expression in protein lysates of SK-Mel-28 cells after 48 h incubation with different concentrations of amlexanox. Western blots show one representative blot of three independent experiments (*n* = 3), and the bar diagrams show the densitometric analysis of all the blots. Hsp90 served as a loading control. (**C**) Terminal deoxynucleotidyl transferase dUTP nick end labeling (TUNEL) staining of the SK-Mel-28 cells after incubation with different concentrations of amlexanox or vehicle for 48 h. The negative control (NC) was processed without the addition of enzyme; cells in the positive control (PC) were subjected to DNase treatment (*n* = 3), Scale Bar: 50 µM.

Mechanisms that are potentially involved in a reduced cell proliferation comprise the induction of necrosis or apoptosis, the blocking of cell cycle progression or the induction of autophagy. The induction of apoptosis or cell cycle blocking was assessed by flow cytometric and Western blot analyses, as well as TdT-mediated dUTP nick end labeling (TUNEL) staining, respectively. The inhibition of IKKε/TBK1 by amlexanox had no impact on the cell cycle stages and did also not increase the cell number in the sub-G1 phase of the cell cycle. Furthermore, the important cell cycle regulating genes p53 and cyclinD1 were not affected by the amlexanox treatment (Figure 3a,b). In the TUNEL staining, we observed no TUNEL-positive cells at 30 µM amlexanox and only a few cells at 50 µM (Figure 3c). Therefore, it is unlikely that the amlexanox-induced antiproliferative effects are due to apoptosis and/or cell cycle inhibition.

**Figure 4:**
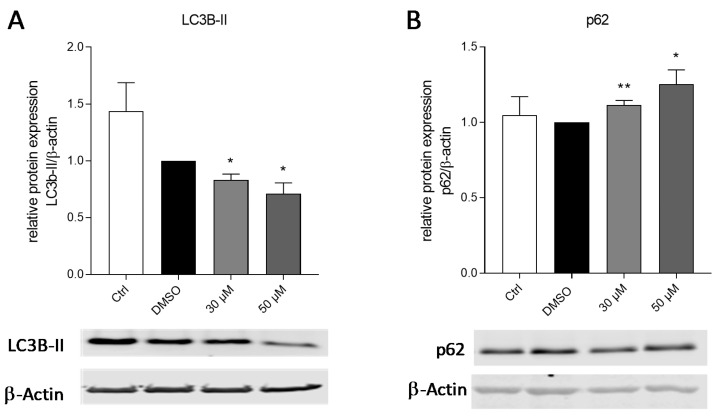
Expression of autophagy markers in SK-Mel-28 cells after amlexanox treatment. (**A**) Light chain 3B (LC3B)-II and (**B**) p62 expression in SK-Mel-28 cells after treatment with amlexanox or vehicle (DMSO). The Western blots show one representative blot out of three independent experiments (*n* = 3); the bar diagrams show the densitometric analysis of all the blots. * *p* < 0.05, ** *p* < 0.01, in comparison to vehicle-treated control cells.

Autophagy is a process of cellular protection but can also influence the proliferation of cancer cells. We investigated autophagic processes by a Western blot analysis of the typical autophagy markers p62 and LC3B-II, which constitute important protein regulators in the formation of the autophagosome. p62 is an autophagy receptor which is degraded during autophagy, while LC3B-I is converted to LC3B-II at the autophagosome membrane. After the amlexanox treatment, we detected a significant downregulation of LC3B-II and an upregulation of p62, indicating that the inhibition of TBK1 and/or IKKε counteracts the induction of autophagy and might thereby inhibit the proliferation of melanoma cells (Figure 4).

### 2.4. The Impact of Amlexanox on Melanoma Cell Migration and Invasion

**Figure 5:**
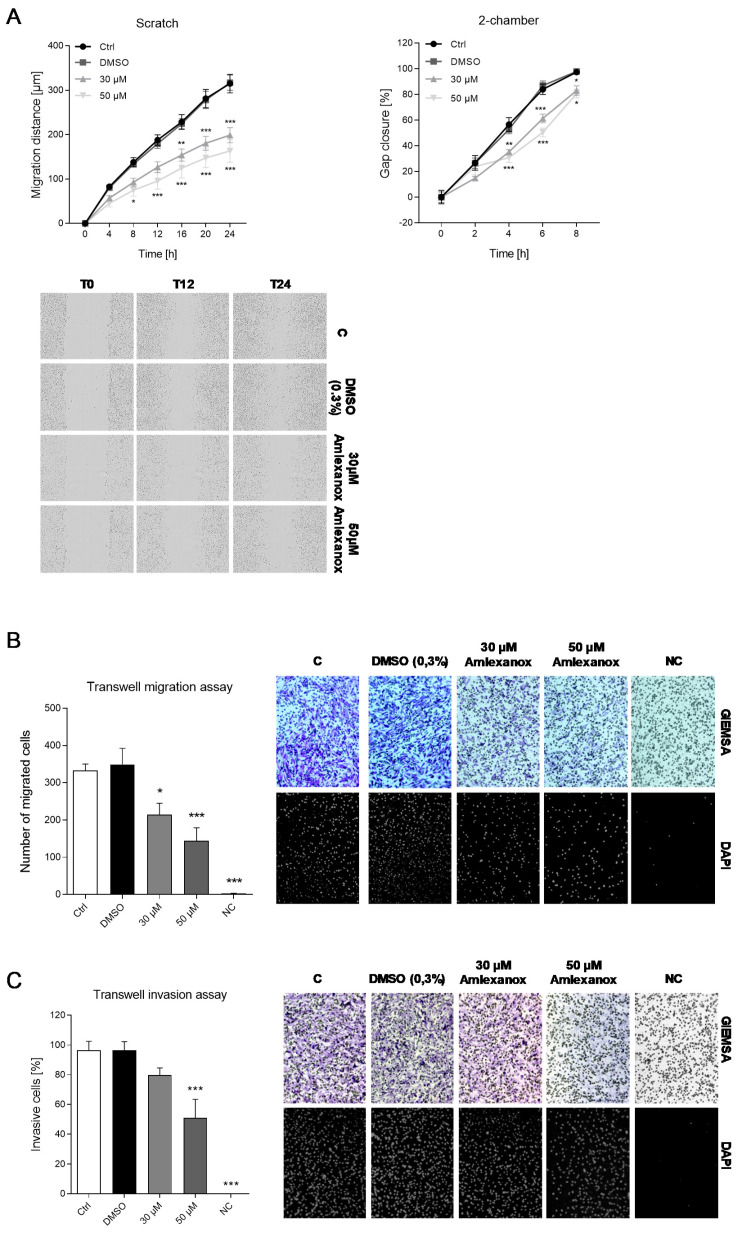
Migration and invasion of SK-Mel-28 cells after amlexanox treatment. (**A**) Scratch and 2-chamber migration assays were used to determine the migration distance of SK-Mel-28 cells in a time-dependent manner. Cells were treated with amlexanox at the indicated concentrations after performing a defined scratch or removing the 2-chamber insert. The images show representative images of the scratch assay (magnification: 10×). The diagrams reveal the analysis of the gap size of all experiments (scratch assay: *n* = 5, 2-chamber assay: *n* = 3). (**B**) Transwell migration assay. The migration of cells through a membrane toward a serum gradient was determined. Serum-free samples served as negative controls. The images show representative images of the transwell migration assay (magnification: 10×). Cells were stained with GIEMSA and 4′,6-Diamidin-2-phenylindol (DAPI). The diagrams reveal the analysis of the number of migrated cells of all experiments (*n* = 5). (**C**) Transwell invasion assay. The migration of cells through a Matrigel-filled membrane toward a serum gradient was determined. Serum-free samples served as negative controls. The images show representative images of the transwell invasion assay (magnification: 10×). The diagrams reveal the analysis of the percentage of migrated cells of all experiments (*n* = 5). * *p* < 0.05, ** *p* < 0.01, *** *p* < 0.001 in comparison to the vehicle-treated control cells.

The release of tumor cells from the basal membrane and their migration toward other tissues as well as invasion into other tissues are hallmarks of tumor progression and metastasis. The melanoma cell migration was assessed by various migration assays. In the scratch assay, a confluent cell layer was wounded by a scratch and then examined over 24 h to determine the cell migration into the gap. The results showed a significant and concentration-dependent inhibition of the cell migration of amlexanox-treated cells. However, since the scratch assay is often associated with wound healing mechanisms due to damage of cell integrity, we performed an additional test which is similar but “damage-free” based on a gap produced by a physical barrier between the two cell layers. Using this assay, we confirmed a significant reduction in cell migration by amlexanox (Figure 5a). These results were affirmed by the transwell cell migration assay. While the control cells with serum-free medium in the lower chamber showed no migration, the untreated and vehicle-treated cells strongly migrated toward the FCS-containing medium. Amlexanox at concentrations of 30 and 50 µM significantly inhibited the migration in comparison with these controls (Figure 5b). The tumor cell invasion was monitored in a transwell setting with an additional layer of Matrigel in the upper transwell chamber. Similar to the migration test, no invasion was determined in the serum-free control, while the untreated and vehicle-treated SK-Mel-28 cells showed a strong invasion into the lower compartment. Amlexanox also inhibited melanoma cell invasion at a concentration of 50 µM; however, the lower concentration of 30 µM was not effective in this assay (Figure 5c).

### 2.5. Regulation of IKKε/TBK1-Dependent Proteins Involved in Melanoma Development

**Figure 6:**
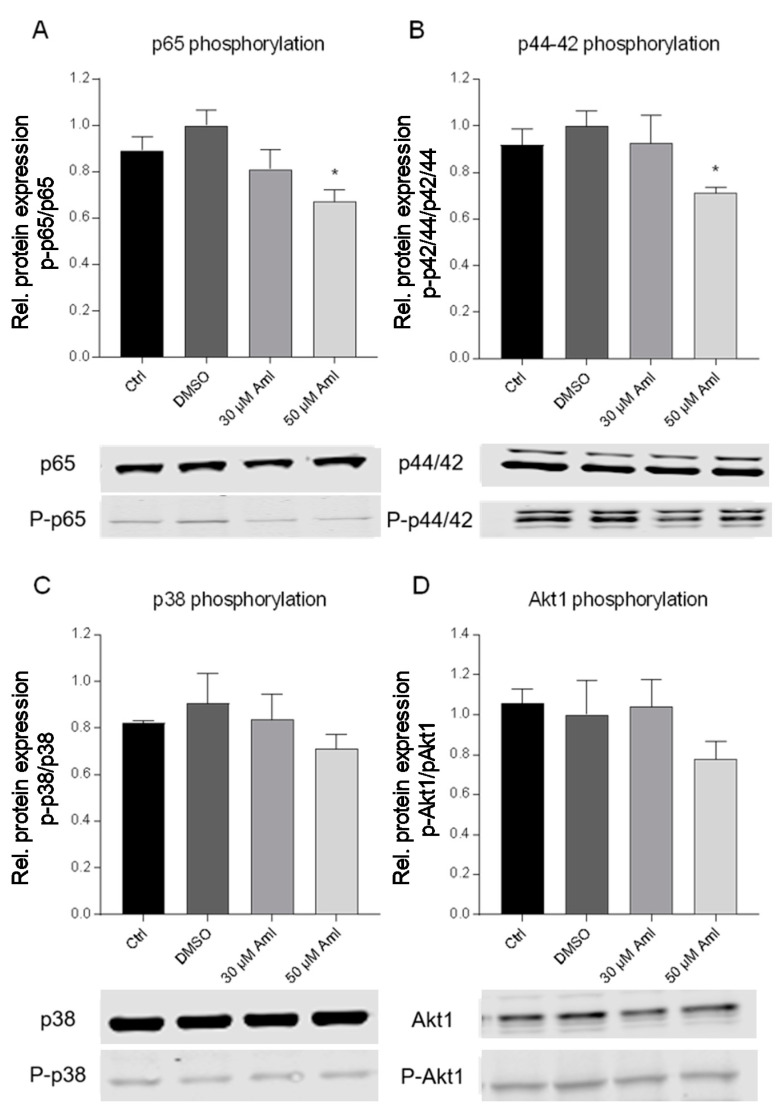
Regulation of genes involved in melanoma development. Activation of p65 (**A**), p42/44 (**B**), p38 (**C**) and Akt1 (**D**) in SK-Mel-28 cells treated with amlexanox at the indicated concentrations, as assessed by Western blot analysis with phospho-specific antibodies. The signals for phosphorylated proteins were normalized to the respective total protein levels and additionally against the loading control β-actin. The blots show a representative result; the diagrams show the densitometric analysis of all the experiments (*n* = 3); * *p* < 0.05 in comparison with vehicle-treated cells.

Several IKKε/TBK1 target genes have already been associated with melanoma initiation and progression. The transcription factor NF-кB is one of their direct targets and is dysregulated in a number of tumors. NF-кB p65 is constitutively active in melanoma, as shown in the Western blot analysis of phosphorylated p65 protein. This effect is slightly enhanced by the treatment of SK-Mel-28 cells with DMSO, but significantly inhibited by 50 µM of amlexanox (Figure 6a). Mitogen-activated kinases (MAPKs) constitute further important regulatory genes in IKKε/TBK1-mediated signal transduction [25,26] which are also constitutively activated in melanoma, thereby controlling the proliferation, survival and invasion of the tumor cells [10,27]. We assessed the phosphorylation of the MAPKs p38 and p42/44 (ERK 1/2) in amlexanox-treated SK-Mel-28 cells. Our results showed no significant effect of amlexanox on p38 phosphorylation but a significantly reduced phosphorylation of p42/44 (Figure 6b,c). Another essential factor constitutively active in melanoma is the serine/threonine kinase Akt1, which is an oncogene leading to tumor progression and also known as an IKKε target. Furthermore, it is known to activate NF-кB p65 downstream of IKKs [10,28]. An amount of 50 µM of amlexanox decreased Akt1 phosphorylation, however this was in a non-significant manner (Figure 6d).

**Figure 7:**
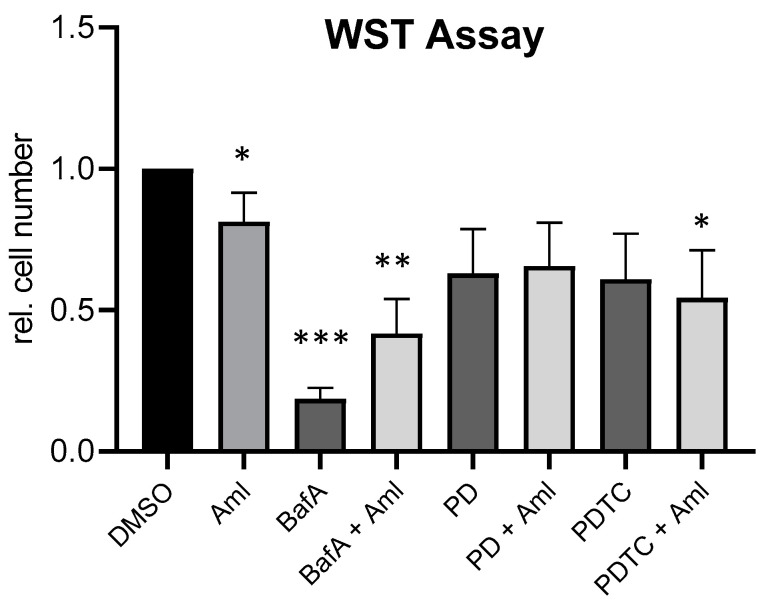
Cell proliferation of SK-Mel-28 cells treated with amlexanox with and without combination with inhibitors for autophagy (bafilomycin A (BafA)), p42/44 (PD98059 (PD) and NF-кB (pyrrolidine dithiocarbamate (PDTC) as assessed by the WST assay. (*n* = 3), * *p* < 0.05, ** *p* < 0.01, *** *p* < 0.001 in comparison to the vehicle-treated control cells.

As a proof-of concept experiment for the above-described results, we performed additional cell proliferation tests using well-known pharmacological inhibitors for the amlexanox-affected mechanism. As an autophagy inhibitor, we used bafilomycin A, p42/44 was suppressed by PD98059 and NF-кB activation by PDTC. The results again showed the inhibitory properties of amlexanox on the proliferation of SK-Mel-28 cells in comparison to the vehicle-treated cells. Bafilomycin A induced a strong reduction in cell proliferation itself but completely suppressed the amlexanox-induced effect. There was rather a slight protective effect by amlexanox. This result might be at least partially due to the fact that bafilomycin A not only affects autophagy but also several other intracellular pathways and processes. PDTC and PD98059 also reduced the cell proliferation by about 35% to 40%, respectively, and diminished the amlexanox-induced reduction in cell proliferation (Figure 7). These results further support a role of autophagy, Erk and NF-кB in the amlexanox-mediated inhibition of tumor cell growth.

### 2.6. Effects of Amlexanox on Tumor Growth in the Nude Mice SK-Mel-28 Xenograft Model

**Figure 8:**
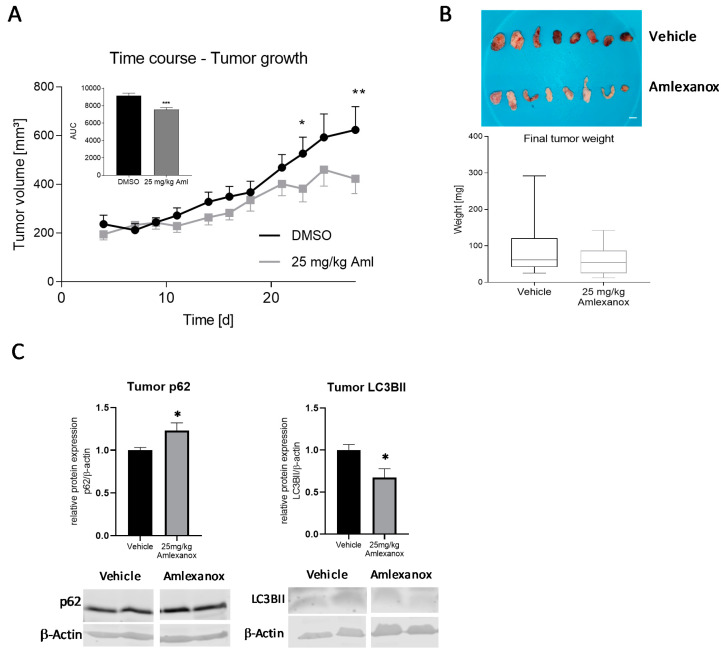
Effects of amlexanox in the nude mice xenograft model. (**A**) Time course of the tumor growth after subcutaneous injection of SK-Mel-28 cells into the flanks of nude mice. The tumor size in mice treated with vehicle (DMSO) (●) or amlexanox (25 mg/kg body weight, p.o.) (■), respectively, was measured every third day until 28 days after injection. The inserted bar graph shows the calculation of the area under the tumor volume vs. time curve (AUC). (**B**) Determination of the tumor weight of the dissected tumor at 28 days (*n* = 12–18 mice/group). Scale Bar: 5mm (**C**) LC3B-II and p62 expression in in the tumors of nude mice after treatment with amlexanox or vehicle (DMSO). The Western blots show two representative samples; the bar diagrams show the densitometric analysis of all samples (*n* = 5–6). * *p* < 0.05, ** *p* < 0.01, in comparison to the vehicle-treated control cells. * *p* < 0.05, ** *p* < 0.01, in comparison to vehicle-treated mice.

To assess the in vivo effect of amlexanox on melanoma development and progression, we applied a xenograft model in nude mice. The mice were subcutaneously injected with SK-Mel-28 cells into both flanks, with one group receiving amlexanox (25 mg/kg body weight p.o., 5 days/week) and a control group treated with the vehicle (DMSO). The melanoma growth was observed over a period of 28 days, and the tumor growth was measured regularly. The establishment of tumors was detectable from day 3 after the injection of the cells. The time curve of further tumor growth indicated that the amlexanox-treated mice revealed a significantly suppressed melanoma growth in comparison to the vehicle-treated mice, which is confirmed by the calculation of the area under the curve (Figure 8a). In addition, the tumor weight at the end of the experiment was determined. The tumors showed a high inter-individual variation and tended to be smaller in amlexanox-treated mice. However, this effect was not significant (Figure 8b). To examine the potential role of autophagy in vivo, we performed a Western blot analysis with protein lysates from the dissected tumors and subjected them to p62 and LC3B antibodies. Similar to the results in cell culture, the p62 protein expression was increased after amlexanox treatment while LC3B-II was upregulated, indicating that the drug also affects the autophagic flux in vivo (Figure 8c).

## 3. Discussion

The IKK-related kinases IKKε and TBK1 are involved in the initiation and progression of several solid tumors. Their inhibition by knock-down or pharmacological drugs has been associated with reduced cancer progression in most cases [29,30]. The impact of TBK1 and IKKε in human malignant melanoma is only scarcely explored. It is known that TBK1, but not IKKε, is overexpressed in mutant NRAS melanoma cells and associated with tumor cell proliferation, migration and invasion, which could be ameliorated by the suppression of TBK1 [21]. Furthermore, a subset of BRAF/MEK-inhibitor-resistant melanoma cells showed sensitivity towards different IKKε/TBK1 inhibitors [22]. In these studies, TBK1 inhibition was suggested as a therapeutic option for the treatment of special melanoma specimens. In addition, TBK1 was involved in NF-кB activation and the subsequent upregulation of PD1-L in a model of the UV light-induced activation of melanoma cells [31]. The present study aimed to further elucidate the impact of pharmacological IKKε and TBK1 inhibition in human malignant melanoma. We showed that IKKε and TBK1 proteins are overexpressed in human melanoma cell lines as well as the metastatic melanoma tissue of patients. Furthermore, both kinases are expressed in the primary human tumor and interestingly not only in tumor cells but also in immune cells, pointing to an additional role of the kinases in the tumor microenvironment, which has been speculated before [32]. The inhibition of IKKε/TBK1 by the administration of amlexanox reduced the proliferation, migration and invasion potential of melanoma cell lines. These observations are in accordance with other studies showing the overexpression of both kinases in cancer and antiproliferative effects in response to their inhibition [33,34,35,36,37], thus confirming the hypothesis that IKKε and TBK1 play important roles in the pathophysiology of various cancer types, including melanoma.

We intended to elucidate the amlexanox-induced mechanism of reduced cell proliferation in melanoma. It has been described many times that melanoma cells are resistant against apoptosis induction, which contributes to the difficulties in the treatment of melanoma patients [38]. In this study, we also showed a lack of apoptosis induction in amlexanox-treated SK-Mel-28 cells. Furthermore, cell cycle distribution and the cell cycle-related proteins cyclinD1 and p53 remained unaltered after amlexanox treatment. Therefore, we assumed that amlexanox might have an impact on autophagy in melanoma cells. The process of autophagy has been associated with cancer several times; however, its distinct role is not clarified due to the fact that it can both suppress and promote cancer progression and metastasis depending on the tumor stage [39,40]. Advanced cancers are often associated with increased autophagic activity, which is suggested to strengthen tumor cells against unfavorable conditions and make them resistant against chemotherapy. The pharmacological inhibition of autophagy might thus constitute a promising approach to treat cancer as well as therapy resistances [41], however only at carefully evaluated stages in tumorigenesis. In melanoma, increased levels of autophagosomes were described, associated with a poor prognosis for the patients [42]. The inhibition of autophagy reduced the development of BRAFV600E mutant melanoma and increased the survival rate of mice in a melanoma model [42,43]. Classical IKK complex subunits have already been associated with the induction of autophagy in an NF-кB-independent manner [44]. The alternative IKKs have also been linked to autophagy, with IKKε as positive regulator in breast cancer cells, thus supporting their proliferation [45], and TBK1 as a contributor to the maturation of autophagosomes and the phosphorylation of the autophagy receptor protein p62 [46], which is overexpressed in several human tumors. In SK-Mel-28 cells and murine tumors, we found an increase in p62 after the administration of amlexanox, which is consistent with elevated levels of p62 in TBK1-deficient mice and the inhibition of autophagy by a combined Janus Kinase (JAK)/TBK1/IKKε inhibitor in pancreatic ductal adenocarcinoma (PDA) [47,48]. Together with the decrease in LC3B-II after amlexanox treatment, our data indicate that TBK1 and IKKε are involved in the pro-survival effects of autophagy in cancer, which might be counteracted by the administration of IKKε/TBK1 inhibitors.

BRAF mutations occur in more than 50% of melanoma, leading to gain of function of BRAF and thereby to the overactivation of the MEK-ERK signaling cascade. Accordingly, BRAF- and MEK- inhibitors gained increasing interest as therapeutic options for melanoma, in many cases in combinatorial therapy [49]. Furthermore, the BRAF alteration is often associated with mutations of PTEN, an inhibitor of the PI3 kinase/Akt1 pathways [50], which in turn causes an increased activation of PI3K/Akt1 and can further enhance melanoma progression. PI3K/Akt1 overstimulation can also enhance NF-кB activation [10,28]. Since previous studies indicated that these proteins also constitute major IKKε/TBK1substrates [4,23,51], we wanted to clarify if the corresponding signaling pathways might contribute to melanoma cell proliferation, migration and invasion and therefore investigated the impact of amlexanox on the activation status of NF-кB p65, Akt1 and the MAP kinases ERK (p42/44) and p38 MAPK. Amlexanox significantly reduced the constitutive phosphorylation of p65 and p42/44 in SK-Mel-28 melanoma cells, indicating that these targets are involved in the anticancerogenous effects of IKKε/TBK1 inhibition, while Akt1 and p38 might only play a minor role in this context. Finally, we investigated the effects of amlexanox on tumor growth in a melanoma xenograft model in nude mice. The oral administration of the drug revealed a significantly reduced tumor growth in comparison to the vehicle-treated mice, which confirms our results from the cell culture. Amlexanox has already shown anti-cancer activity in different tumor types—e.g., breast cancer [52,53], prostate cancer [54] and glioblastoma [55]—And is now also identified as a promising therapeutic option in melanoma. Since amlexanox constitutes an approved drug with already confirmed pharmacological safety and only minor side effects, it might be promising to consider its re-purposing for the treatment of melanoma. However, due to the important roles of IKKε/TBK1 in the immune system, the effects of their inhibition on pathogen defense and immune function must be carefully monitored.

In summary, our data indicate that IKKε and TBK1 might contribute to growth and metastasis of malignant melanoma. Their inhibition is associated with reduced cell proliferation, migration and invasion, probably by decreased autophagy and the reduced activation of melanoma-relevant proteins. Due to these properties, the inhibition of their activity by amlexanox can suppress tumor growth. In conclusion, the repurposing of amlexanox might be considered as a promising option for the therapy of melanoma and is possible in patients who are resistant to current therapeutic options.

## 4. Materials and Methods

### 4.1. Drugs

Amlexanox (2-Amino-7-(1-methylethyl)-5-oxo-5H-[1] benzopyrano [2–3-b] pyridine-3-carboxylic acid), used as specific small molecule inhibitor of TBK1 und IKKε, was purchased from Cayman Chemical (Ann Arbor, MI, USA). Amlexanox was dissolved at a concentration of 10 mg/mL in 100% DMSO. For cell treatment, a dilution series was performed to gain end concentrations of 10, 20, 30 and 50μM to allow the incubation of cells with an equal DMSO concentration of maximal 1%. Pyrrolidine dithiocarbamate (PDTC) was purchased from Sigma (Munich, Germany) and was dissolved in DMSO at a concentration of 100mM. PD98059 and Bafilomycin A were from Cell Signaling Technologies (Boston, MA, USA). Bafilomycin A was dissolved at 2.5 µM and PD98059 at 10mM, both in DMSO.

### 4.2. Animals

Foxn1 nude mice were purchased from Envigo (Venray, AN the Netherlands) at the age of 4 weeks. They were housed under constant conditions, including a 12 h light-dark cycle and a room temperature of 24 ± 0.5 °C, with food and water ad libitum. Tumor cell inoculation started at the age of 6 weeks. In all the experiments, the European ethic guidelines for investigations in conscious animals were obeyed and the procedures were approved by the local Ethics Committee for Animal Research (Regierungspräsidium Darmstadt, 28 August 2017, FK/1092). All efforts were made to minimize animal suffering and to reduce the number of animals used. The experiments were performed by an observer blinded for the treatment.

### 4.3. Nude Mouse Xenograft Model

Foxn1 nude mice under 2% isoflurane anesthesia were injected subcutaneously with 5 × 10^6^ SK-Mel-28 human melanoma cells in 100 µL of PBS into the left and right flank. The mice were treated orally with amlexanox (25 mg/kg body weight) [56] or vehicle (DMSO) for 28 d, starting at the day of tumor cell injection. For this purpose, the drug and vehicle were applied 5 days/week on commercially available corn flakes in a 10% sucrose solution. The tumor volume was determined at day 4, 7, 9, 11, 14, 16, 18, 21, 23, 25 and 28 with a digital caliper. The volume of the tumor was calculated with the equation “V = π/6 * 1.69 * (length * width)^1.5^”. On day 28, the animals were sacrificed and the tumors were dissected and weighed (wet weight).

### 4.4. Cell Culture

A375M and SK-Mel-28 human melanoma cells were used, which both carry a homozygous BRAF^V600E^ mutation. The A375M cells were from Wellcome Trust (London, Great Britain), and the SK-Mel-28 cells were purchased from CLS (Eppelheim, Germany). These cell lines were cultivated in DMEM medium with 10% FCS in a humidified atmosphere at 37 °C and 5% CO_2_. HERMES1 human melanocytes were purchased from Wellcome Trust (London, Great Britain) and cultivated in RPMI 1640 medium (Thermo Fisher Scientific, Schwerte, Germany) containing 220 nM tetradecanoyl phorbol acetate (TPA), 220pM Cholera Toxin (CT), 11 nM Endothelin1 (EDN1), 10 ng/mL human stem cell factor (hSCF) (all Sigma, Munich, Germany) and 10% FCS (Thermo Fisher Scientific, Schwerte, Germany) at 37 °C and 10% CO_2_.

### 4.5. Cell Proliferation Assays

The determination of the cell proliferation and potential toxicity of amlexanox was performed with the sulforhodamine B (SRB) (Sigma, Munich, Germany) and the WST assay, respectively. The SRB assay is used to determine cell density based on the analysis of the cellular protein content, while the WST assay detects the activity of cellular dehydrogenases in living cells. For both assays, 5 × 10^4^ SK-Mel-28 or A375M cells were seeded in 500 µL of medium into each well of a 24-well tissue culture plate. After 24 h, the cells were treated with fresh medium containing the vehicle (DMSO) and 10, 20, 30 and 50 µM amlexanox for 48 h, respectively. For combinatorial analyses, 30 µM amlexanox was used with and without combination with 100 µM of PDTC (Sigma, Munich, Germany), 10 µM of PD98059 and 2.5 nM of Bafilomycin (both Cell Signaling Technologies (Boston, MA, USA)), respectively. All the inhibitors were also applied singularly at the indicated concentrations. At the end of the cultivation period, the medium was replaced by fresh medium containing the WST-1 reagent (Roche Diagnostics (Sigma-Aldrich, Munich, Germany)) for an incubation time of 90 min at 37 °C to allow the metabolization of WST-1 to formazan. The absorption of the medium was then determined photometrically at 450 and 620 nm in a Tecan microplate reader. For the SRB assay, the cells were fixed with 5% trichloroacetic acid (TCA) for 1 h at 4 °C. The plates were washed seven times with H_2_O and then dried for 1 h at 60 °C. The staining of cellular proteins was performed for 30 min at RT with sulforhodamine B (SRB) (Sigma, Darmstadt, Germany) at a concentration of 0.4% in 1% acetic acid (Roth, Karlsruhe, Germany). The plates were washed five times with 1% acetic acid and then dried again for 1 h at 60 °C. SRB was dissolved in 10 mM Tris pH 10.5 and the extinction of the stained supernatant was measured photometrically at 546 nm.

### 4.6. Migration and Invasion Assays

The migration capacity of SK-Mel-28 cells with and without the amlexanox treatment was assessed using three different types of assays based on scratch migration, closing a gap between two chambers or passing a filter membrane.

#### 4.6.1. Scratch Assay

An amount of 5 × 10^4^ cells were seeded into each well of an IncuCyte ImageLock 96-well plate (IncuCyte, Essen, Germany) with and without the addition of amlexanox or DMSO, respectively. After 24 h at 37 °C, the medium was replaced by starving medium (2% FCS), and the confluent cell layer was scratched with an IncuCyte 96-pin wound making tool. The subsequent migration of the cells into the gap was observed and documented with the IncuCyte ZOOM software every 4 h for 24 h. The data were exported as the width of the cell-free area. For the calculation of the migration distance, the equation “dt_x_ = (dt_0_ − dt_x_)/2”, where dt_x_ is the distance at time x, was used.

#### 4.6.2. 2-Chamber Assay

Since the scratch assay leads to damage of the cellular integrity and is therefore often used as a wound healing assay, we used an additional assay which is not based on the injury of the cells. An amount of 3 × 10^4^ SK-Mel-28 cells were seeded into 2-chamber culture inserts of 24-well black µ-plates (Ibidi, Gräfeling, Germany) with and without the vehicle or amlexanox treatment. After 24 h incubation at 37 °C, the inserts were removed and the medium was replaced by starving medium (2% FCS). The migration of the cells into the gap was observed under the Image Xpress micro Confocal High-Content Screening System (Molecular Devices, Biberach a.d.R., Germany) at 37 °C by taking pictures of two defined positions per gap every 2 h for a total of 36 h. The width of the cell-free gap was measured in every picture and the change was analyzed over time (MetaXpress software, Molecular Devices, Biberach a.d.R, Germany).

#### 4.6.3. Transwell Migration Assay

In this assay, transwell filters with a pore size of 8 µM were inserted in the wells of a 24-well plate. The lower chamber wells were then filled with 750 µL of culture medium. For the negative control, FCS-free medium was used. An amount of 5 × 10^4^ SK-Mel-28 cells were disseminated in 300 µL of FCS-free medium into the upper chambers with and without the vehicle or amlexanox in different concentrations. After 48 h of incubation at 37 °C, the cells on the lower side of the filter membrane were fixed with 4% PFA for 2 min and 100% methanol for 20 min and then stained with 1 mL GIEMSA solution for 15 min at RT and subsequently with 800 µL of DAPI solution for 2 min. The number of migrated cells was assessed under a microscope (Zeiss AxioVision40 4.7.2, Zeiss, Germany). Pictures of the three different areas of the membrane were taken with 10× magnification, and the number of cells was counted with the help of ImageJ’s automated cell counting tool (imagej.nih.gov). The mean of the three areas was taken as the cell count.

#### 4.6.4. Invasion Assay

The experimental set-up of the invasion assay is similar to the transwell migration assay, except that the transwell inserts are filled with Matrigel as an additional barrier. An amount of 100 µL of diluted Matrigel (BD bioscience; San Jose, CA, USA) (0.3 mg/mL in serum-free medium) was placed in the upper chamber and allowed to fix for 24 h at 37 °C. After that, the protocol corresponds exactly to the transwell assay. The invasive capacity was calculated with “Number of invasive cells/ Number of migrated cells × 100”.

### 4.7. Autophagy Assay

An amount of 5 × 10^5^ cells were disseminated in 3 mL of medium into a 6 cm cell culture dish and incubated for 24 h at 37 °C. The medium was changed to an FCS-free medium and the relevant treatments were added to the dishes. Then, the cells were again incubated with the addition of amlexanox or vehicle for 24 h at 37 °C. The cells were harvested and the proteins were isolated. The autophagic activity was assessed by a Western blot analysis of LC3B-II and p62 expression.

### 4.8. Western Blot Analysis

The cells were washed with 0.1 M phosphate buffered saline (PBS), scraped with a rubber policeman and collected in 1.5 mL tubes. After short centrifugation, the pellet was resuspended in PhosphoSafe Extraction Buffer (Merck, Darmstadt, Germany) containing protease inhibitor (1 mM Pefabloc SC, Alexis Biochemicals, Lausen, Switzerland) and kept at room temperature for 3 min. Then, the cell lysate was centrifuged at 14,000 rpm for 10 min at 4 °C in an Eppendorf centrifuge, and the supernatant was stored at −20 °C until further analysis.

The proteins (20 µg) were separated electrophoretically by 10% SDS-PAGE and then transferred onto nitrocellulose membranes by semi-dry-blotting. To control the quality of the transfer, all the blots were stained with Ponceau red solution. The membranes were blocked for 60 min at room temperature in Odyssey blocking reagent (LI-COR Biosciences, Bad Homburg, Germany) diluted 1:2 in 0.1 M PBS, pH 7.4. Afterward, the blots were incubated overnight at 4 °C with primary antibodies against IKKε (1:500, Active Motif, Carlsbad, CA, USA), p62 (1:250, Abcam, Cambridge, UK), TBK1, LC3B, p-Akt1, Akt1, pp65-S536, p65, pp44/42, p44/42, pp38 or p38 (all the antibodies 1:250, Cell Signaling Technology, Boston, MA, USA) in Odyssey blocking reagent diluted 1:2 in 0.1% Tween 20 in 0.1 M PBS. After washing three times with 0.1% Tween 20 in 0.1 M PBS, the blots were incubated for 60 min with an IRDye 700-conjugated secondary antibody (Molecular Probes (Eugene, OR, USA, 1:10.000 in blocking buffer diluted 1:2 in 0.1% Tween 20 in 0.1 M PBS). After rinsing in 0.1% Tween 20 in 0.1 M PBS, protein-antibody complexes were detected with the Odyssey Infrared Imaging System (LI-COR Biosciences). β-actin (37 kDa) and Hsp 90 (both 1:1.200, Sigma, Munich, Germany) were used as the loading controls. A densitometric analysis of the blots was performed with the Image Studio Lite Software (LI-COR Biosciences, Bad Homburg, Germany).

### 4.9. Cell Cycle Analysis

The influence of IKKε inhibition on the cell cycle distribution of human melanoma cells was determined by fluorescence-activated cell sorting (FACS). An amount of 6 × 10^5^ SK-Mel-28 cells were seeded into 10 cm cell culture dishes and attached for 24 h at 37 °C. To synchronize the cells in the G1 phase of the cell cycle, the medium was then replaced by serum free medium for 24 h. Thereafter, cells were treated for 24 h in normal cell culture medium with addition of amlexanox and DMSO, respectively, and then harvested and fixed in 70% ethanol (*v*/*v* in PBS) for at least 24 h at −20 °C. For FACS analysis, the cells were centrifuged for 5 min at 3000 rpm at 4 °C. The supernatant was discarded, the fixed cells were permeabilized with 0.25% Triton X-100 for 5 min at 4 °C and stained with propidium iodide (20 µg/mL in PBS containing 0.2 mg/mL RNaseA (Qiagen, Hilden, Germany)) (Sigma, Darmstadt, Germany) in the dark for 30 min on ice. An amount of 1 × 10^6^ cells/sample were counted using an LSR Fortessa SORP 5-Laser 2B 5V 3R 5YG CST FACS device and visualization and documentation were performed with the FACSDiva 6.1.3 software (BD Bioscience, San Jose, CA, USA). G1, S and G2/M as well as the subG1 and subG2 fractions were quantified using the FlowJo 10.1 software (BD Bioscience, San Jose, CA, USA) and manual gating.

### 4.10. TUNEL Staining

TdT-mediated dUTP nick end labeling (TUNEL) staining was performed with an in situ cell death detection kit purchased from Roche (Sigma, Munich, Germany) as recommended by the manufacturer. In brief, SK-Mel cells were incubated for 48 h with amlexanox at the indicated concentrations. Then, the cells were rinsed with PBS and fixated with 4% paraformaldehyde. Blocking was performed with 3% H_2_O_2_ and lysis with 0.1% Triton X-100. Afterwards, TUNEL labeling started for 60 min with labeling solution and then 30 min with POD converter. As peroxidase substrate, 3, 3 -diaminobenzidine (DAB) was added for 5 min, the slides were rinsed 3 times with PBS and then the staining was analyzed by microscopy (Zeiss Primovert, Jena, Germany).

### 4.11. Multiplex Immunofluorescence Analysis

Paraffin-embedded primary melanoma tissue samples were provided by UCT Frankfurt. Tumor sections (4 µm) were stained and analyzed using the Opal staining system according to the manufacturer’s instructions (PerkinElmer, Rodgau, Germany). The following antibodies were used for staining: IKKε (Active Motif, Carlsbad, CA, USA), TBK1 (Cell Signaling Technologies (Boston, MA, USA)), CD45 (Abcam, Cambridge, UK), PD1 (Abcam, Cambridge, UK), CD3 (Ventana, Roche Diagnostics, Mannheim, Germany). Corresponding secondary HRP-coupled antibodies were anti-mouse IgG-HRP and anti-rabbit IgG-HRP (both GE Healthcare, Freiburg, Germany). Nuclei were counterstained with DAPI and slides were mounted with Aqua-Poly/Mount (Polysciences, Hirschberg, Germany). The slides were imaged at 4× and 20× magnification using the Vectra3 imaging software, and the images were analyzed using the inForm2.0 Software (PerkinElmer, Rodgau, Germany).

### 4.12. Data Analysis

The statistical evaluation was performed with GraphPad Prism 7 for Windows. The data are presented as mean ± SEM. The data were either compared by a univariate analysis of variance (ANOVA) with subsequent Student’s *t*-tests employing a Bonferroni α-correction or Dunnett’s correction for multiple comparisons, by a repeated-measures two-way ANOVA or by a Student’s *t*-test. For all the tests, a probability value *p* < 0.05 was considered as statistically significant.

## Abbreviations

Akt1	Serine/threonine-protein kinase 1, protein kinase B
BRAF	V-raf murine sarcoma viral oncogene homolog B1
DMSO	dimethylsulfoxide
IFN	interferon
IRF	Interferon regulatory factor
IKKε	IκB Kinase epsilon
LC3	Microtubule-associated proteins 1A/1B light chain 3B
MAPK	Mitogen activated kinase
NF-кB	Nuclear factor kappa B
NRAS	Neuroblastoma RAS viral oncogene homolog
PD1	Programmed cell death protein 1
PTEN	Phosphatase and tensin homolog
TBK1	Tank binding kinase 1
TUNEL	Terminal deoxynucleotidyl transferase dUTP nick end labeling
